# Structural and optical properties of Nd:YAB-nanoparticle-doped PDMS elastomers for random lasers

**DOI:** 10.1038/s41598-021-95921-8

**Published:** 2021-08-19

**Authors:** Antsar R. Hlil, Jyothis Thomas, Yalina Garcia-Puente, Jean-Sebastien Boisvert, Bismarck C. Lima, Ando Rakotonandrasana, Lauro J. Q. Maia, Amirhossein Tehranchi, Sebastien Loranger, Anderson S. L. Gomes, Younes Messaddeq, Raman Kashyap

**Affiliations:** 1 Fabulas Laboratory, Department of Engineering Physics, École Polytechnique Montréal, Station Centre-ville, P.O Box 6079, Montreal, QC H3C 3A7 Canada; 2 Département de Chimie, Faculté des Sciences et de Génie Pavillon Alexmoura Vachon, Université Laval, 1045, avenue de la Médecine, Quebec, G1V 0A6 Canada; 3 Centre d’Optique, Photonique et Laser, Université Laval, 2375 Rue de la Terrasse, Quebec, QC G1V 0A6 Canada; 4 Center for Telecommunications Studies, Pontifical Catholic University of Rio de Janeiro, Rio de Janeiro, Brazil; 5 Grupo Física de Materiais, Instituto de Física, Universidade Federal de Goiás-UFG, Campus II, Av.Esperança 1533, Goiânia, GO 74690-900 Brazil; 6 Departamento de Física, Universidade Federal de Pernambuco, Recife, PE Brazil; 7 Fabulas Laboratory, Department of Electrical Engineering, École Polytechnique Montréal, Station Centre-ville, P.O Box 6079, Montreal, QC H3C 3A7 Canada

**Keywords:** Chemistry, Materials science, Nanoscience and technology, Optics and photonics, Physics

## Abstract

We report the structural and optical properties of Nd:YAB (Nd_x_Y_1−x_ Al_3_(BO_3_)_4_)-nanoparticle-doped PDMS elastomer films for random lasing (RL) applications. Nanoparticles with Nd ratios of x = 0.2, 0.4, 0.6, 0.8, and 1.0 were prepared and then incorporated into the PDMS elastomer to control the optical gain density and scattering center content over a wide range. The morphology and thermal stability of the elastomer composites were studied. A systematic investigation of the lasing wavelength, threshold, and linewidth of the laser was carried out by tailoring the concentration and optical gain of the scattering centers. The minimum threshold and linewidth were found to be 0.13 mJ and 0.8 nm for x = 1 and 0.8. Furthermore, we demonstrated that the RL intensity was easily tuned by controlling the degree of mechanical stretching, with strain reaching up to 300%. A strong, repeatable lasing spectrum over ~ 50 cycles of applied strain was observed, which demonstrates the high reproducibility and robustness of the RL. In consideration for biomedical applications that require long-term RL stability, we studied the intensity fluctuation of the RL emission, and confirmed that it followed Lévy-like statistics. Our work highlights the importance of using rare-earth doped nanoparticles with polymers for RL applications.

## Introduction

There is significant research interest in flexible structured photonic devices based on polydimethylsiloxane (PDMS)^1^. In addition to the field of materials science^2,3^ flexible photonic devices have also raised interest for bio-medical applications, such as lab-on-a-chip (LOC), electronic skins, and medical prosthetics^4,5^. Because of their promising features such as high flexibility and good mechanical and chemical stability, PDMS-based devices could be suitable as mechanical sensors. They have also been used as tunable phase masks for writing fiber Bragg gratings^6^ and for tunable diffractive optic elements^7^. The flexibility and stretchability of these polymers also makes them excellent materials for random laser (RL) applications, in which lasing is obtained via multiple scattering in a disordered structure^8–10^. In the bulk^11^, RL emission is obtained via multiple scattering of light emitting in random directions with very low temporal or spatial coherence^12^. Recent studies on polymer-based random lasers have mainly focused on their low lasing threshold and flexibility to realize compact and disposable visible-wavelength lasers^13^, which are appropriate for sensing applications^14^. However, they generally suffer from issues related to the stability of dye based RL systems^15^, which could be overcome using appropriate nanomaterials, such as ZnO nanoparticles^16^. To improve the performance of polymer based RLs, more efficient pathways to design stable systems using rare-earth-doped nanoparticles are necessary. The use of PDMS polymer allows the ease of material fabrication and processability in view of potential applications along with the use of rare earth materials.

A very promising application is its use as a photon source for speckle free imaging by taking advantage of its brightness due to the laser emission as well as and its low spatial coherence^17,18^. Also RLs find applications in opt microfluidics^19^, optical batteries^20^, cancer diagnostics^21^ etc. A recent work by N. Caselli shows that the engineering of coupled RLs sets the basis for building structures with potential to function as optical neural networks^18^.

Previously, we demonstrated the potential application of inorganic Nd:YAB nanoparticles with PDMS for RL applications, and the material system demonstrated high durability^22,23^. This work provides a further and deeper continuation of Ref.^22^, which demonstrated random lasing with PDMS composite films which contain Nd:YAB (Nd_0.8_Y_0.2_Al_3_B_4_O_12_) nanoparticles around 1064.5 nm with pulsed nanosecond excitation at 808 nm and 532 nm. In the current work, we have systematically investigated the lasing wavelength, threshold, and linewidth of the laser by tailoring the concentration and optical gain of the scattering centres. One of the underlying questions related to the physics of random lasing is the influence of the density of scattering centers on the random lasing properties. This is because the mean free path of a photon is strongly affected by the correlation length, Lc^24^. For this reason, tailoring the concentration and optical gain of scattering centers is of primary interest. We managed to alter the dopant density within the PDMS-based composite to dynamically tune the distribution of the scatterers and gain medium by stretching the PDMS medium. Inherently stable and long-lasting lasing was realized in this novel elastomeric composite by exploiting the dependence of the lasing emission on the scatterer density, making them suitable for biomedical applications. We also studied the intensity fluctuation of the RL emission and confirmed that it follows Lévy statistics.

## Results and discussion

### Structural and morphological characterization

The composition of the PDMS films and the polymer composites with Nd:YAB nanoparticles are given in Table 1.

**Table 1:** Composition of the polymer composite with Nd:YAB nanoparticles.

Polymer composite	Composition PDMS/curing/Nd_x_Y_1−x_Al_3_(BO_3_)_4_ -(3%)
PDMS-Sylgard	Polymer/curing/0.5 g/0.05 g
PC0.2 (X = 0.2)	PDMS/curing/Nd_0.2_Y_0.8_Al_3_(BO_3_)_4_ (0.1 g/0.01 g/0.03 g)
PC0.4 (X = 0.4)	PDMS/curing/Nd_0.4_Y_0.6_Al_3_(BO_3_)_4_ (0.1 g/0.01 g/0.03 g)
PC0.6 (X = 0.6)	PDMS/curing/Nd_0.6_Y_0.4_Al_3_(BO_3_)_4_ (0.1 g/0.01 g/0.03 g)
PC0.8 (X = 0.8)	PDMS/curing/Nd_0.8_Y_0.2_Al_3_(BO_3_)_4_ (0.1 g/0.01 g/0.03 g)
PC1.0 (X = 1.0)	PDMS/curing/Nd_1.0_ Y_0.0_Al_3_(BO_3_)_4_ (0.1 g/0.01 g/0.03 g)
PC0.0 (X = 0)	PDMS/curing/Y_1.0_Al_3_(BO_3_)_4_ (0.1 g/0.01 g/0.03 g)

The incorporation of nanoparticles into PDMS (host polymer) as well as the structural difference between samples with and without Nd:YAB nanoparticles were investigated by Raman spectroscopy.

**Figure 1:**
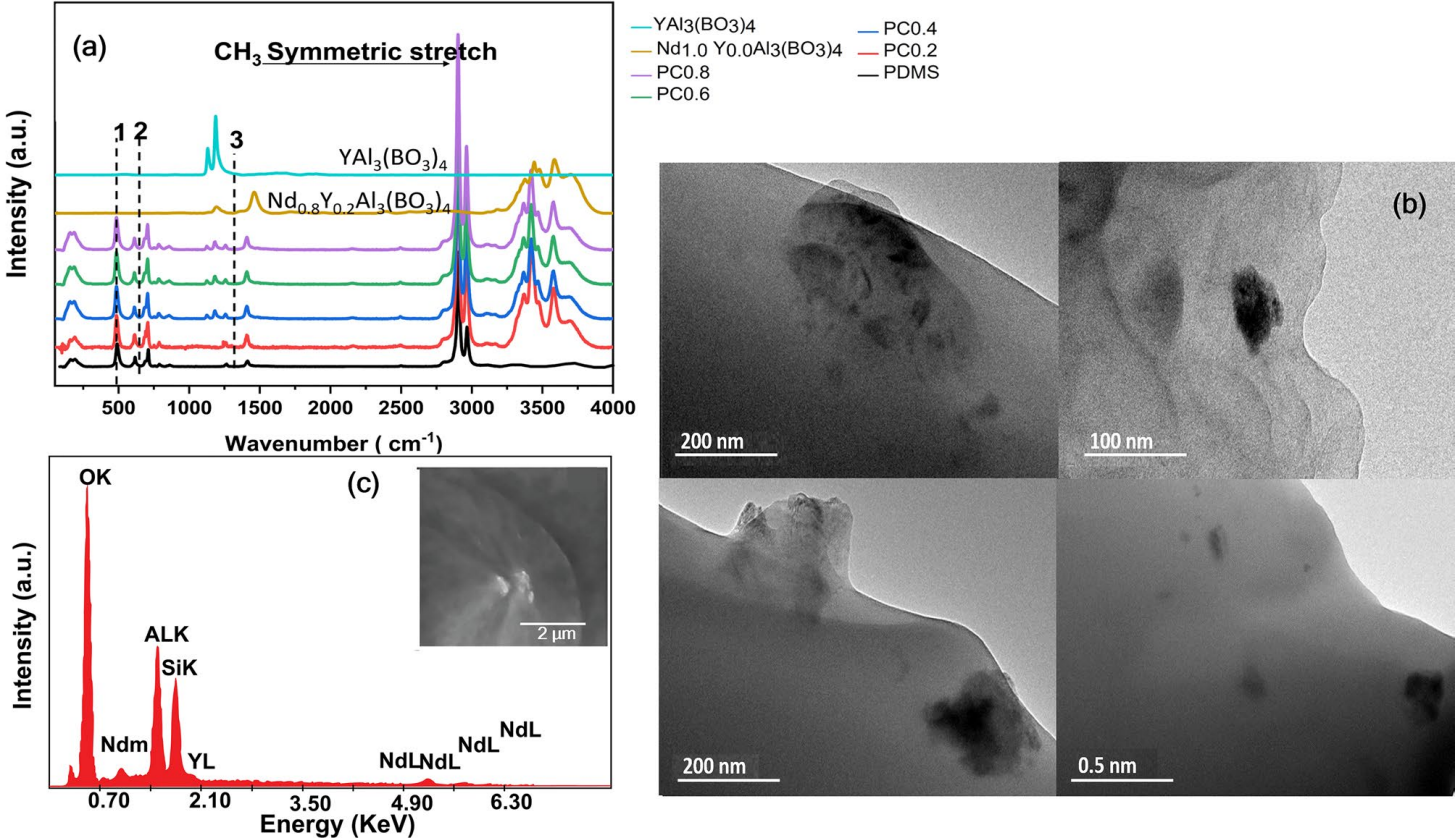
(**a**) Raman spectra of pristine PDMS (black curve) and PDMS nanocomposite with Nd_x_Y_1−x_Al_3_(BO_3_)_4_ (PCx) with a 633 nm pump laser. Also shown are nanoparticles and YAl_3_(BO_3_)_4_ without PDMS (dark yellow and light blue lines respectively). Noted in the figures are (1) Si–O–Si Symmetric stretch peak at 487 cm^−1^, (2) the Si–CH_3_ symmetric rocking at 687 cm^−1^, and (3) the Si–CH_3_ symmetric bending 1260–1400 cm^−1^, (**b**) TEM Images of PDMS composites with ultrafine particle incorporated with PDMS, (**c**) energy-dispersive X-ray analysis of PC0.8. The inset shows an SEM image of the sample with 5 µm scale.

The Raman spectrum of PDMS without the Nd_x_Y_1−x_ Al_3_(BO_3_)_4_ particles is shown in Fig. 1a in black. The typical vibrational modes for silicon, carbon, oxygen, and hydrogen bonds can be observed; these include symmetric stretching (Si–O–Si, 487 cm^−1^), symmetric rocking (Si–CH_3_, 614 cm^−1^), (Si–C, 707 cm^−1^), asymmetric stretching (Si–C) overlaps with asymmetric rocking (CH_3_) at 789 cm^−1^, and symmetric stretching (CH_3_) at 2965 cm^−1^ and at 2906 cm^−1^. These assignments are in close agreement with those found in the literature^25^.

The Raman spectra of Nd_x_Y_1-x_Al_3_(BO_3_)_4_-nanoparticle-doped PDMS composites are also shown in Fig. 1a. New bands appearing at 1191 and 1463 cm^−1^ for the PDMS + nanoparticles composite samples could be attributed to the stretching of trigonal BO_3_ groups^26^, while the bands in the range 3300–3800 cm^−1^ can be attributed to the presence of Nd_x_Y_1−x_Al_3_(BO_3_)_4_. From Fig. 1a, it can be deduced that the intensity of bands depends on the Nd_x_ ratio in the composites incorporated in the PDMS polymer.

From comparison of the Raman spectra of Nd_x_Y_1-x_Al_3_(BO_3_)_4_ particles in Supplementary Information Fig. S1 and the polymer composite (PC), it can be clearly seen that the vibrational band intensities, which represent changes in the sample, decrease and disappear more slowly for the PDMS-composite (PC) compared with the pure polymer. The changes in the intensity of all the characteristic vibrational bands are significant. From these measurements, it can be further noted that the lack of any new major vibrational bands indicates that no new Raman active chemical species or side products are created.

The thermal behavior of composites was measured via DSC, and the results did not show any significant difference in the glass transition temperature between the pure PDMS and the composite samples, as reported in Table 2. This indicated that the introduction of nanoparticles with strong polymer interaction did not affect the glass transition temperature. The crystallization point was not clearly observed, which is in close agreement with literature reports where PDMS chains were reported to be unable to crystallize when they are adsorbed or in high proximity to the filler (particles)^27,28^.

**Table 2:** Thermal properties of polymer composite with Nd:YAB.

Polymer composite (Nd_x_)^a^	DSC		TGA	
	Tg^b^ (°C)	∆Cp^c^ J/(g K)	5% (weight lost)^d^	Total% (lost at 600 °C)^e^
PDMS-Sylgard	− 123.1	0.167	396	− 57.1
Y_1.0_Nd_0_Al_3_(BO_3_)_4_ PC0.0	− 123.0	0.157	397	− 45.59
PC0.2	− 122.1	0.153	408	− 27.18
PC0.4	− 122.0	0.154	403	− 28.1
PC0.6	− 121.8	0.151	400	− 30.7
PC0.8	− 121.6	0.151	400	− 30.7
PC1.0	− 121.6	0.151	403	− 28.8

^a^Polymer composite containing Nd concentrations of Nd_x_Y_1–x_Al_3_B_4_O_12_ with x = 0.2, 0.6, 0.8, and 1.0.

^b,c^Obtained by DSC under a helium atmosphere with heating rate of 10 K/min.

^d,e^Obtained by TGA under 25 mL/min stream nitrogen atmosphere with constant heating ramp of 10 °C/min.

The morphology and the cross section of the Nd_0.8_Y_0.2_Al_3_(BO_3_)_4_ particles embedded in the PDMS films were analyzed at different magnifications using transmission electron microscopy (TEM) (Fig. 1b) and scanning electron microscopy (SEM). The morphology of the powder particles embedded into the polymer were observed using a high-resolution transmission electron microscope (TEM and HR-TEM modes) on a JEOL model JEM 2010 operating at 200 keV and is shown in Fig. 1b.

The composition of the polymer composite was determined via energy-dispersive X-ray analysis (EDX) (Fig. 1c), and the composite were found to contain all the elements of the NPs and PDMS^29^. From the SEM images shown in the inset of Fig. 1c it can be seen that the Nd nanoparticles were randomly distributed in the PDMS substrate and the broad dispersion and present a larger particle quantity between 100 and 300 nm. The SEM image with 1 µm magnification is provided in Supplementary Information Fig. S3.

The heat capacity ∆Cp data listed in Table 2 were determined from the DSC curves given in the supplementary materials (Supplementary Information Fig. S4). The data did not show any significant differences in the ∆Cp for the polymer composites.

In the glassy state, a high degree of cooperativity between the chain segment is required, which is associated with a high energy barrier in the early stages of transition. As the temperature increases, the molecular motion increases, hence, the degree of cooperativity decreases, which consequently led to a decrease in the effective activation energy.

The glass transition for pure PDMS and PDMS with nanoparticles were similar, which indicated that the presence of nanoparticles that had a strong interaction with the polymer did not affect the glass transition temperature. TGA was used to evaluate the thermal stability of the prepared polymers composites. The thermal decomposition of the PC samples was evaluated at different percentages of weight loss (5% and at the maximum), and the results are summarized in Table 2. Supplementary Information Figure S5 shows the thermogravimetric analysis, i.e., TGA curves of the PDMS-Sylgard polymer and the composites with Nd:YAB, which were heated to 800 °C at a constant rate of 10 °C min^−1^ under a 25 mL min^−1^ nitrogen stream. The PDMS displayed weight‐loss stages located in the 220–450 and 450–580 °C ranges, which can be attributed to PDMS degradation.

### Random lasing emission

**Figure 2:**
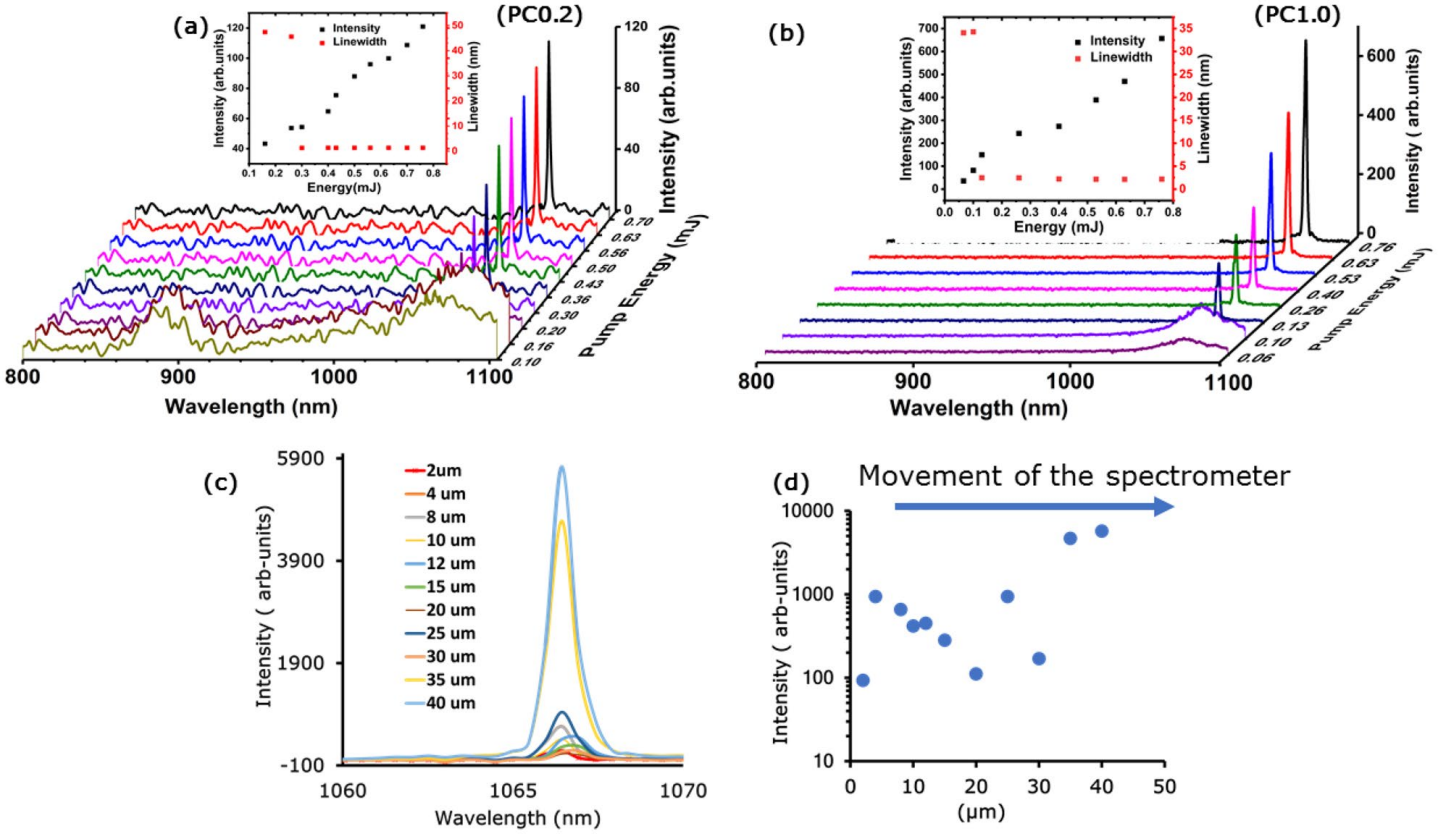
(**a**) The emission spectra obtained from the Nd_0.2_Y_0.8_Al_3_(BO_3_)_4_ polymer composite, and the threshold is 0.30 mJ. On further increasing the pump energy, a narrow peak emerges in the emission spectra with a linewidth of 1.31 nm. The inset shows the FWHM (red circles) and maximum emission integral intensity (black circles) vs. pump energy, (**b**) emission spectra obtained from the Nd_1.00_Al_3_(BO_3_)_4_ polymer composite, with an emission threshold of 0.13 mJ. On further increasing the pump energy, narrow peaks emerge in the emission spectra with a linewidth of 2.21 ± 0.1 nm. The inset shows the FWHM (red squares) and maximum emitted intensity (black circles) vs. pump energy. The higher noise in Figure is due to the different scaling which is normalized to the highest peak in each figure, (**c**) the lasing spectrum of PC0.8 as the spectrometer is moved in same direction (µm), which increases or decreases depending on the position of the spectrometer. The (**d**) shows the change in the laser intensity with varying position of the spectrometer.

The RL emission spectra of the PC0.2 polymer composite sample is shown in Fig. 2a for different excitation energies, which range from below to well above the RL threshold. The obtained linewidth, i.e., the full width at half maximum (FWHM) of the emission spectra, for the same sample composition as a function of pump energy is shown in the inset of Fig. 2a. A Gaussian fit was used to calculate FWHM. An example fitting for Nd_1.0_Y_0.6_Al_3_B_4_O_12_ polymer composite for ASE and RL are provided in supplementary information (SI3—Fig. S6). The emission linewidth is strongly reduced, ranging from 48 nm just before lasing begins (amplified spontaneous emission (ASE)) to 1.3 ± 0.5 nm (spectrometer bandwidth limited), as the excitation pump energy is increased from below to well above the excitation threshold (~ 0.30 mJ). A detailed analysis of PC0.2 and PC1 can be found in Supplementary Information Figs. S8 and S11.

The PC1 sample is shown in Fig. 2b and it displays similar behavior but with a much higher emission intensity and lower threshold. It can be noted that ASE generated at ~ 900 nm becomes negligible at higher Nd nanoparticle ratios. The increase in the Nd^3+^ concentration is correlated with the RL wavelength because it corresponds with the maximum of the gain at 1064 nm. Additional spectra for samples PC0.4 to PC0.6 are available in the supplementary material (Supplementary Information Figs. S9, S10). The shift of the random lasing emission spectra change in the linewidth as well as the lasing threshold depends on the Nd^3+^ concentration and correlates to the RL wavelength because it corresponds to the maximum of the gain curve. Tuning of the laser in the case of the narrow bandwidth of Nd is possible in terms of directionality, ASE and lasing. There is only limited wavelength tunability due to the narrow gain bandwidth of Nd. A detailed study on the absorption and photoluminescence spectra of the gain material were already provided in previous paper (Ref.^30^) and is reproduced in the SI (Fig. S7).

The RL emission spectra varied with observation angle, which is because the RL device emits in multiple directions with various intensities. The lasing spectra for sample PC0.8 observed while moving the spectrometer in the same direction is shown in Fig. 2c. The lasing intensity in different directions, with a constant pump power, can increase or decrease depending on the position of the spectrometer which demonstrates the multi-directional operation of the RL as shown in Fig. 2d. The lasing parameters for each sample are described in Table 3.

**Table 3:** The obtained lasing wavelength, threshold, and linewidth for the polymer composites (PC0.2–PC1.0).

Sample	Lasing wavelength (nm)	Threshold (mJ)	Lasing linewidth (nm)	Lifetime (µs)
PC0.2	1066.4 ± 0.50	0.30	1.3	40
PC0.4	1065.9 ± 0.50	0.26	0.9	26
PC0.6	1065.9 ± 0.50	0.20	0.9	23
PC0.8	1064.0 ± 0.50	0.20	0.8^17^	19
PC1.0	1066.9 ± 0.50	0.13	2.2	18

The transition from spontaneous emission to the lasing regime was demonstrated by the decay time measurements of the 4F3/2 level. The measured lifetimes are described in Table 3 and detailed results are available in the supplementary material (Supplementary Information Fig. S12). Above the threshold, the pulse width was determined to be 10 ns. The obtained lifetimes for all the polymer composites are in good agreement with previous reports for the corresponding nanocrystalline powders^30^. As expected, the lifetimes of the polymer composites are almost similar to the nanocrystalline powders^30^. Due to ion-ion interaction and energy transfer between ions, the lifetime decreases as the doping concentration is increased^31^.

**Figure 3:**
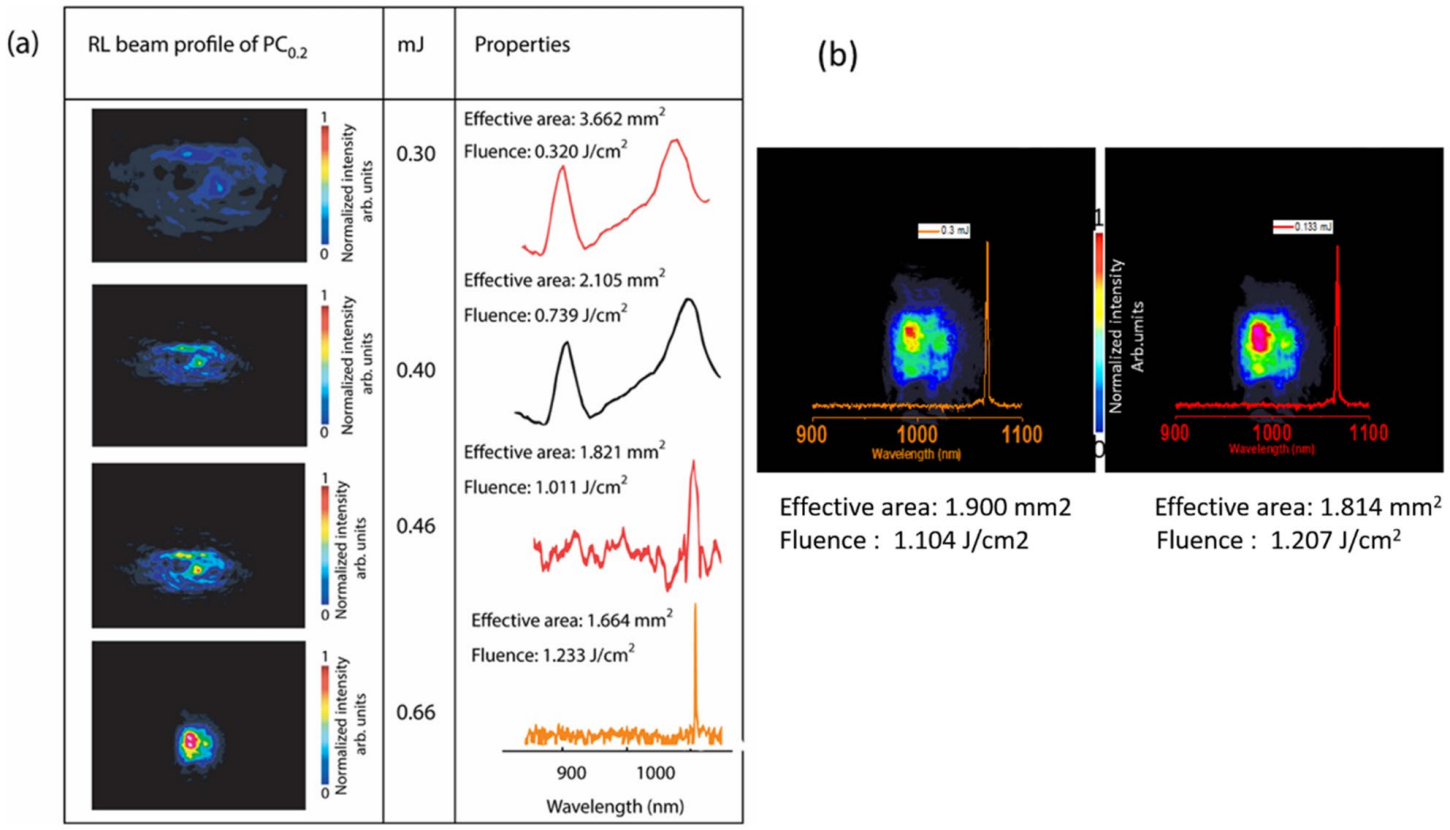
(**a**) Properties of the observed RL beam profile for polymer composite PC0.2 at different excitation energies, (**b**) the random laser beam profile of sample PC1.0 and lasing emission at pump energies of 0.16 and 0.3 mJ.

The RL beam profile of PC0.2 along with the emission spectra and properties are provided in Fig. 3a. The beam profiles were obtained using a coherent Laser Cam HR beam profiler (Coherent). Firstly, the lasing emission was verified using the spectrometer, which was then replaced with the beam profiler to record the image. The color bars represent relative intensities from low to high.

When the excitation energy was increased from 0.3 to 0.66 mJ the effective beam area reduced from 3.6 to 1.6 mm. The observed RL beam profile for sample PC1.0 at an input pump energy of 0.16 and 0.30 mJ are shown in Fig. 3b. The effective area of the beam crossection and the fluence were 1.900 mm^2^ and 1.104 J/cm^2^, respectively, using a pump energy of 0.16 mJ. However, above the threshold, the effective area and fluence were measured to be 1.814 mm^2^ and 1.207 J/cm^2^, respectively.

### Stretching, stability and repeatability studies on polymer composites

The RL emission intensity as well as the wavelength tunability were investigated by stretching the polymer composite. This was performed by increasing the distance between two translation stages holding each edge of the polymer strip. The percentage stretching is defined by $${\raise0.7ex\hbox{${\Delta L}$} \!\mathord{\left/ {\vphantom {{\Delta L} L}}\right.\kern-\nulldelimiterspace} \!\lower0.7ex\hbox{$L$}} \times 100$$ i.e., ratio of change in the length to the original length of the polymer composite.

Laser decrease and stop during the stretching process due to mode hoping. As shown in Fig. 1, the nanoparticle has no specific orientation. Stretching process changes the orientation of the polymer chains, not the nanoparticles and this can lead to change in the intensity. We have explored the number of scatterers/unit volume by stretching of the polymer host and demonstrated that the random phases of the scatter and also the variability in the gain from the different particles of rare earth element, Neodymium, influences not only the wavelength of emission (albeit limited as the RE used does not have a wide gain bandwidth), but also lasing, and the direction of emission. The sum of the phases of the scattered radiation determines if the RL is going to lase. Hence, at some strain values, lasing does not occur.

**Figure 4:**
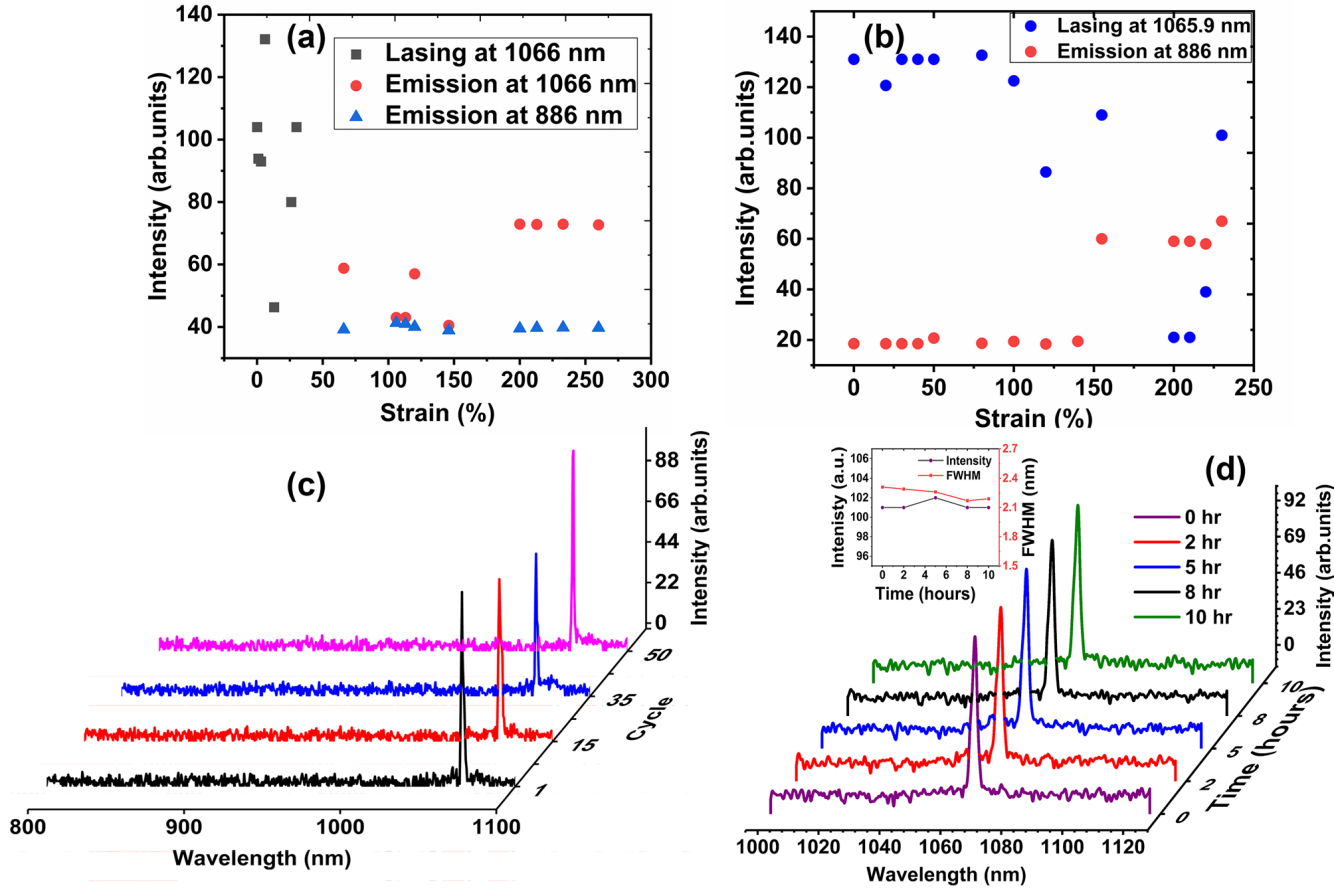
(**a**) The spectroscopic response intensity of the PC0.2 device for different strain values up to 260% with 532 nm laser excitation. Each point in Figure is an average of the peak spectral power density over different strain values. Lasing ceases for strain values above ~ 60% and only amplified spontaneous emission (ASE) can be observed instead, with one new peak appearing at 886 nm, (**b**) spectroscopic response intensity of the PC0.4 RL device for different strains up to 230%, (**c**) RL emission of PC1.0 by applying a maximum strain of 100% for several cycles, (**d**) the PC1.0 RL emission as a function of time of continuous operation, the inset shows the intensity and linewidth (FWHM) of the lasing emission as a function of time of continuous operation.

The RL emission of the PC0.2 device at 1066.4 ± 0.5 nm was sustained up to 30% of stretching. However, lasing stops at strain values > 60% and amplified spontaneous emission (ASE) occurs instead, with one new peak appearing at 886 nm (Fig. 4a). This is because during each stretching event, the phase of the scattered light changes and phase matching can be lost. In addition, the stretching process changes the orientation of the nanoparticles in the polymer. Furthermore, at certain strain values the amplitude of the RL emission reduces, however, lasing does not stop.

The linewidth (FWHM) of the lasing at 1066.4 nm was measured to be 1.31 nm for 0–30% strain. The ASE bandwidth at 1066 nm was 61.22 nm for 60–250% of strain. The ASE peak at 886 nm has a linewidth of ~ 30 nm. For the present composition, lasing could not be observed above 50% strain.

A new emission line at 886 nm appeared for PC0.4 at strain > 155%, but only amplified spontaneous emission (ASE) is observed. The new emission line (ASE) appeared at 886 nm for PC0.4 at strain > 155%. The emergence of this peak is due to the 4F_3/2_ to 4I_9/2_ fluorescence transition^32^. We could observe this only in PC0.2 and PC0.4. In all other samples, the lasing peak at ~ 1064 nm (due to 4F3_/2_ to 4I_11/2_ transition) was dominant. The spectroscopic response of the RL device for different strains is shown in Fig. 4b, which represents the intensity versus strain at 1064 and 886 nm.

A perfectly uniform and adiabatic stretching will change the defects’ position scaling but will not change the orientation, which will lead to a linear phase change, hence a shift in the emitted wavelength^17^. However non-uniform change (which is more likely to happen) will change the random distribution of defect, hence changing the lowest threshold localization mode, leading to a mode-hop. This is why we expect the emission bandwidth to change.

If a mode hop occurs, then the threshold, linewidth and emission direction may change somewhat as the random media has a large number of possible modes with different quality factors. However, as a general rule of thumb for the sample measured and which our data supports, stretching the material lowers the lasing threshold, possibly through a better phase matching condition, and is detailed in Table 3. Since lower pump power is now closer to threshold, the linewidth increases slightly according to the Schwalow–Townes relation^33^. Also increasing the concentration of Nd could increase the gain of the system and reduce the threshold. A detailed statistical study of the intensity fluctuation versus strain with emission bandwidth as well as the mechanical properties (for e.g.: tensile strength) on the tuning of the laser wavelength is being studied in detail and will be reported in the future article. A detailed statistical study focusing on the effect of stretching as well as the mechanical properties (for e.g.: tensile strength) on the tuning of the laser wavelength is being studied in detail and will be reported in future.

The average thickness of all samples we used are ~ 0.3 ± 0.1 mm. Although we do not have a fixed cavity in the random laser, the mean free path of the photons depends on the particle density. Therefore, by stretching the sample, the effective area under illumination increases, leading to a decrease in effective density. As the mean free path is inversely proportional to particle density, it increases in the present case.

For PC0.6, the RL emission at 1065.95 ± 0.5 nm was sustained up to 270% strain, while it could be sustained up to 300% stretching for PC1. However, the intensity reduced suddenly above 200% strain. Detailed RL spectra at various strains is available in the Supplementary Information Figs. S13–S16. The different lasing regimes can be attributed to the random scatterer density that influences the random lasing behavior with stretching. In addition, the RL emission wavelength and intensity could be gently restored when the strain was released from 100 to 0%, which demonstrates good repeatability. This was estimated by measuring the RL spectrum during each cycle of applied strain, i.e., continuously applying a strain up to 100% and then releasing it to 0% strain for ~ 50 cycles at a pump energy of 0.30 mJ. The observed highly repeatable and strong lasing spectra shown in Fig. 4c demonstrates that our polymer composite has excellent stability even after repeated stretching. Organic materials are unfortunately known for their poor stability over time, and hence, we studied the lasing operation over a long period. The stability of the Nd_1.0_Al_3_(BO_3_)_4_ composite was investigated by monitoring the RL emission with continuous laser excitation for 10 h at an excitation energy of 0.30 mJ, which is far above the threshold (Fig. 4d). The intensity and linewidth variation are shown in Fig. 4d and demonstrate that the sample had good stability in terms of its emission intensity and FWHM. The combination of stable inorganic Nd:YAB nanoparticles with PDMS enabled extended stability of the material system for long-term experiments and measurements without degradation^17^.

There are many advantages for stretchable random lasers. Firstly, we can tune the laser intensity, wavelength or threshold just by stretching the lasing material^34^. Secondly, it can be useful for developing flexible structure photonics devices, particularly for use in display technology as well as optical sources for biomedical applications, wearable devices etc.^35^.

It should be noted that each polymer composite was found to exhibit unique RL emission characteristics. Finally, we compared the lasing performance of all the polymer composites and found that PC1.0 was the most suitable composition for RL applications. This is the most striking finding of this study, i.e., that sample PC1.0, which contains a large amount of scattering and gain particles, exhibits a lower lasing threshold (0.1 mJ) than other samples. In addition, the measurements show that PC1.0 has a lifetime of 18 µs (lower than the lifetimes of the other polymer composites), which is desirable because it can facilitate the lasing process. Along with these properties, the observed highly repeatable and stable random lasing spectrum, even with repeated stretching of the polymer, allowed us to conclude that PC1.0 is the best RL media among the investigated composites. For PC1.0, the RL emission at 1066.9 ± 0.5 nm was sustained up to 270% of stretching, which was not observed for other samples. The observed results are consistent with previous reports on Nd:YAB (Nd_x_Y_1−x_Al_3_(BO_3_)_4_) nanocrystalline powders^30^.

We performed experiments on a novel RL device to study its intensity fluctuation and spectral correlation via analysis of the distribution of the maximum emission intensities. Our previous work on PC0.8^22^ was the first experimental study of the probability distribution function (PDF) of the emitted intensities in a SERL (stable elastomer random laser) system. Lévy-like statistics of the intensities and photonic spin glass behavior were also demonstrated in this open cavity random laser.

For the PC0.8 polymer composite, on increasing the pump energy from below to above the RL threshold, a transition from a Gaussian statistical regime to a Lévy-like one was observed, followed by a return to the Gaussian statistical regime (Supplementary Information Fig. S17). The PDF of the emission intensities was determined by studying the RL output intensity fluctuations. A replica symmetry-breaking above the RL threshold was observed and the photonic spin-glass behavior of the RL emission was established^22^.

## Conclusion

A near infrared (NIR) random laser composed of Nd:YAB nanoparticles in an elastomeric polymer was demonstrated. Lasing at a wavelength of ~ 1065 ± 1 nm was observed from the Nd:YAB nanoparticles under 532 nm excitation. It was verified that the Nd:YAB nanoparticles and the gain medium act as scattering centers, while the PDMS polymer acts as a highly stable host substrate. Additionally, the polymer composites could be stretched by up to 300% to control the emission. Benefitting from their unique properties, such as a lower lasing threshold and highly repeatable and stable RL spectrum, the polymer composites PC0.8 and PC1.0 were found to be excellent candidates for RL applications compared to the other investigated samples. In particular, they are suitable for the development of flexible lasing devices, which are still technologically challenging and expensive. One potential application is in the field of bio-photonics, where the stability of the RL is of importance, and where our SERL could possibly emulate flexible biomaterials, with PDMS being a highly bio-compatible material. In principle, based on the requirements for random laser device requirements, the optical properties of different rare-earth doped RLs can be tuned by varying the RE concentration and the pumping wavelength.

## Materials and methods

### Synthesis of nanocrystal powders

Nd_x_Y_1−x_Al_3_(BO_3_)_4_ nanocrystal powders, with Nd ratios x = 0.2, 0.4, 0.6, 0.8, and 1.0 were synthesized via the polymeric precursor method, as previously described^30^. Briefly, in a typical reaction, aluminum nitrate (Al(NO_3_)_3_·9H_2_O) and neodymium nitrate (Nd(NO_3_)_3_·6H_2_O) were dissolved in aqueous citric acid (C_5_O_7_H_8_) solution at room temperature. The resultant mixture was then added to an aqueous solution containing D-sorbitol (C_6_O_6_H_14_) and boric acid (H_3_BO_3_). Dried resin was produced by polymerization of the blend at 150 °C, with the molar ratio maintained at 3:1 for citric acid and elements (boron with metal), while the d-sorbitol/citric acid ratio was 2:3. The dried resin was first calcinated at 400 °C for 24 h, and then annealed at 700 °C for a further 24 h, and finally heated at 1100 °C for 5 min under abundant oxygen supply.

## Fabrication of the PDMS/Nd_x_Y_1-x_ Al_3_(BO_3_)_4_ composites

**Figure 5:**
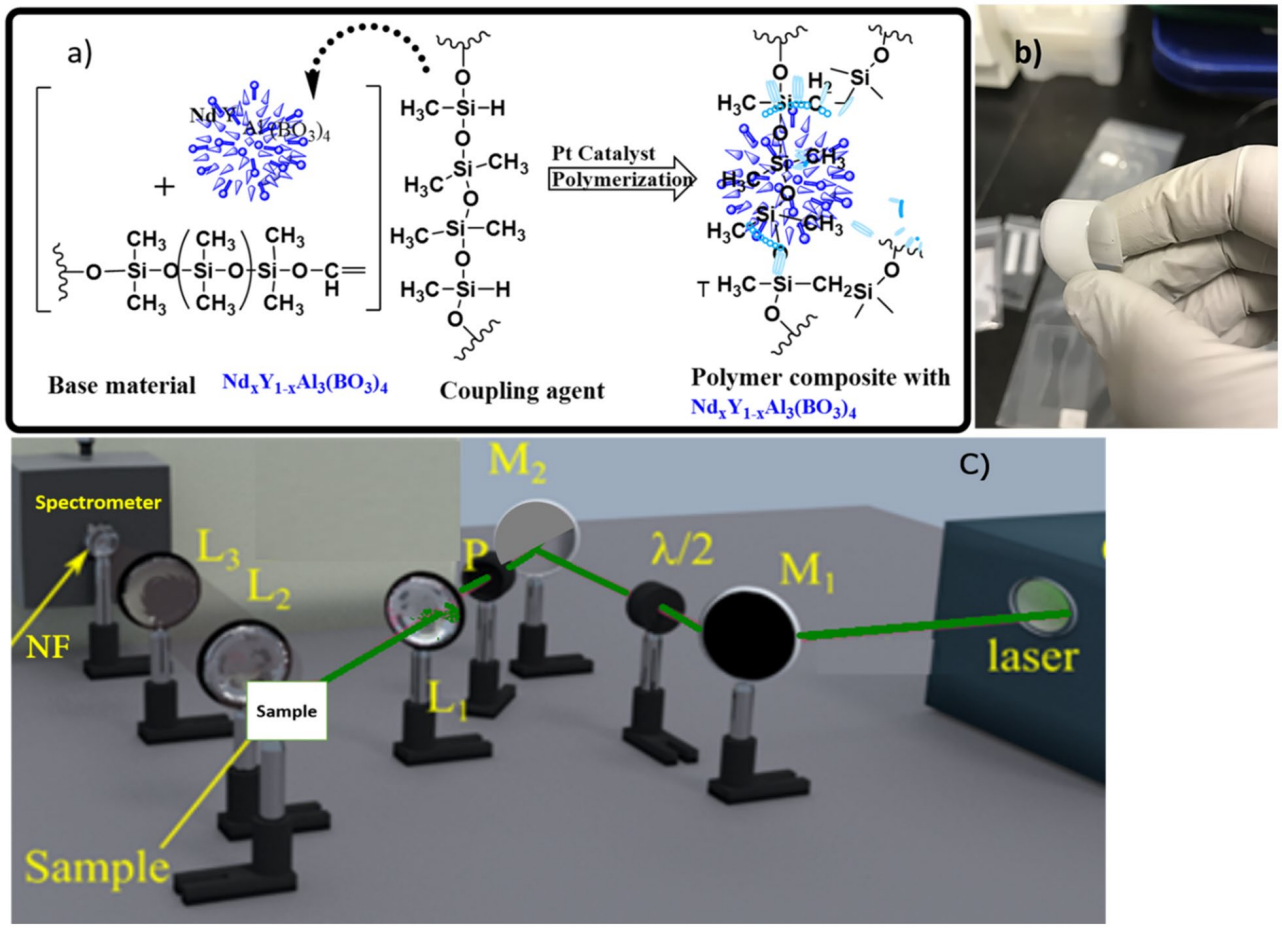
(**a**) The composition of the polymer composite, and (**b**) THE synthesized flexible polymer composite, (**c**) Experimental setup used to excite the samples and collect the RL emission. The source of excitation is a Nd:YAG laser at 532 nm. The beam intensity is controlled through a half-wave plate (HWP) and a polarizer (P) and is focused on the sample by lens L_1_. The light signal is collimated by lens L_2_ and focused on the spectrometer entrance by lens L_3_. The signal is detected using a spectrometer.

The PDMS (Sylgard 184) was obtained from Ellsworth Adhesives Canada. PDMS along with its respective curing agent (Component B) at a ratio of 10:1 was used with the Nd:YAB nanoparticles (PC) for preparation of the polymer composites shown in Fig. 5a. The PDMS prepolymer containing di-vinyl-terminated (Sylgard-184: Base material) was poured into an acrylic container in which 3% (w/w) of the previously prepared Nd_x_Y_1−x_Al_3_(BO_3_)_4_ particles were dispersed with extensive mixing and subsequent ultrasonication for 15 min. The blend was kept under vacuum for 30 min to remove bubbles produced during the mixing process. Next, a curing agent (Component B) containing Si–H groups along with a platinum catalyst was added to the blend, and it was further sonicated for 1–2 min. Flexible films (Fig. 5b) were then obtained by either: spin-coating the polymer composites on pre-cleaned glass slides with an initial speed of 100 rpm for 30 s, followed by 200 rpm for 30 s, and finally, the samples were cured at 90 °C for 1 h; or, by pouring the blend into a 5 × 1 cm mold of thickness (1 mm, 0.3 mm, 0.2 mm) followed by curing in an oven at 90 °C for 1 h. The dispersion of nanoparticles in the PDMS monomer prior to polymerization was a better approach for avoiding nanoparticle aggregation^18^.

## Characterization

The structural and optical properties of the polymer composites were investigated and characterized. Diffuse reflectance spectra (DRS) in the range of 250–1500 nm were collected using a UV/Vis/NIR spectrophotometer (PerkinElmer: Lambda WB 1050) equipped with the Praying Mantis attachment.

The Raman spectra were recorded with a Renishaw in Via spectrometer coupled with a Leica DM2700 microscope. A back-scattering geometry was used in the frequency range of 50–4000 cm^−1^. The excitation light source was a horizontally polarized laser operating at 633 nm and a Syncerity detector (Model 1024X256-OE, Horiba). The Raman spectroscopy measurements were performed with a 100× objective (Olympus MPL—NA = 0.90).

Thermogravimetric measurements were performed using a Mettler-Toledo Q500 TGA/DSC (Columbus, Ohio, USA) instrument, and 5–8 mg samples placed in alumina crucibles were heated up to 800 °C at a constant rate of 10 °C min^−1^ under a 25 mL min^−1^ nitrogen stream.

Differential scanning Calorimetric (DSC) curves were recorded using Netzsch, DSC–404F3 Pegasus in-line with a liquid nitrogen cooling unit. For the measurements, 20 mg samples packed in aluminum pans with highly sensitive type E sample carrier were introduced in a silver furnace with a heating rate of 10 K/min under a helium atmosphere. The glass transition temperatures (T_g_) are reported based on the temperature at the middle of the thermal transition from the second heating scan.

The RL emission spectra of five polymer composites were measured under identical experimental conditions at room temperature using a pulsed Nd:YAG laser (150 Hz, 10 ns, *λ* = 532 nm), delivering a maximum energy of 0.76 mJ. Programing automates data collection using labVIEW2019SP1 software.

The emission signal was collected and analyzed using an Ocean Optics USB4000 spectrometer (spectral resolution ∼ 0.5 nm) (Fig. 5c).

The beam profiles of the RL were acquired using a coherent Laser Cam HR beam profiler.

The emission decay curves upon excitation at 532 nm were acquired using a fast oscilloscope (Tektronix). A Thorlabs SM05PD1B photodiode was used to record the signal.

## Supplementary Information

Supplementary Information.
