# Supplementary material for: Structural and optical properties of Nd:YAB-nanoparticle-doped PDMS elastomers for random lasers
Source: Sci Rep. 2021 Aug 19;11:16803. doi: 10.1038/s41598-021-95921-8 (PMC8377032; doi:10.1038/s41598-021-95921-8)
Supplement: Supplementary file 1 — Supplementary Information. [file 41598_2021_95921_MOESM1_ESM.pdf]

Supporting Information

Structural and optical properties of Nd:YAB-nanoparticle-doped PDMS elastomers for random lasers

Antsar R. Hlil[1,2,3*], Jyothis Thomas[1], Yalina Garcia-Puente[1], Jean-sebastien Boisvert[1], Bismarck C. Lima[4], Ando Rakotonandrasana[1], Lauro J.Q. Maia[5], Amirhossein Tehranchi[1], Sebastien Loranger[1], Anderson S. L. Gomes[6], Younes Messaddeq [2,3] and Raman Kashyap [1,3,7]

[1]*Fabulas Laboratory, Department of Physics Engineering, École Polytechnique Montréal, P.O Box 6079, Station Centre-ville, Montreal, QC, H3C 3A7, Canada.*

[2]*Département de chimie, Faculté des sciences et de génie Pavillon Alexmoura Vachon, 1045, avenue de la Médecine, Université Laval, Québec, G1V 0A6, Canada*

[3]*Centre d'Optique, Photonique et Laser, 2375 Rue de la Terrasse, Université Laval, Québec, QC, G1V 0A6, Canada.*

[4]*Center for Telecommunications Studies, Pontifical Catholic University of Rio de Janeiro, Rio de Janeiro, Brazil*

[5]*Grupo Física de Materiais, Instituto de Física, Universidade Federal de Goiás-UFG, Campus II, Av.Esperança 1533, 74690-900, Goiânia, GO, Brazil.*

[6]*Departamento de Física, Universidade Federal de Pernambuco, Recife-PE, Brazil*

[7] *Fabulas Laboratory, Department of Electrical Engineering, École Polytechnique Montréal, P.O Box 6079, Station Centre-ville, Montreal, QC, H3C 3A7, Canada.*

[*]antsar.hlil@polymtl.ca

SI1. Raman spectra of the $Nd_xY_{1-x}Al_3(BO_3)_4$ nanopowders

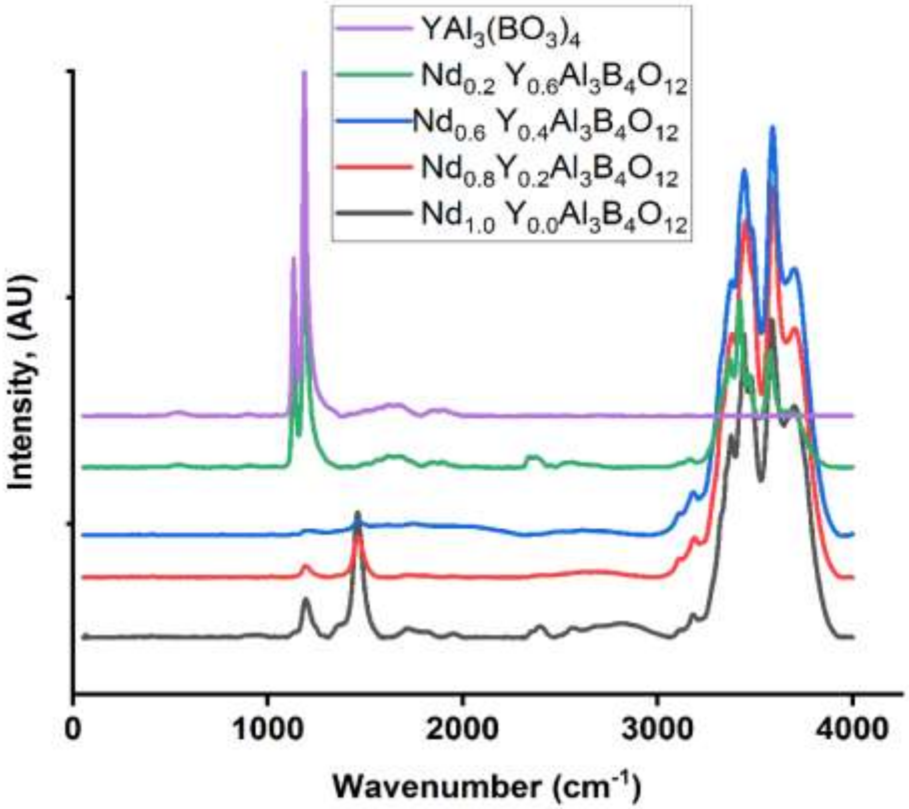

Fig. S1: Raman spectra of the $Nd_xY_{1-x}Al_3(BO_3)_4$ nanopowders (x between 0 and 1) from 200 to 4000 cm^{-1}, under excitation at 633 nm from a He-Ne laser

SEM Images

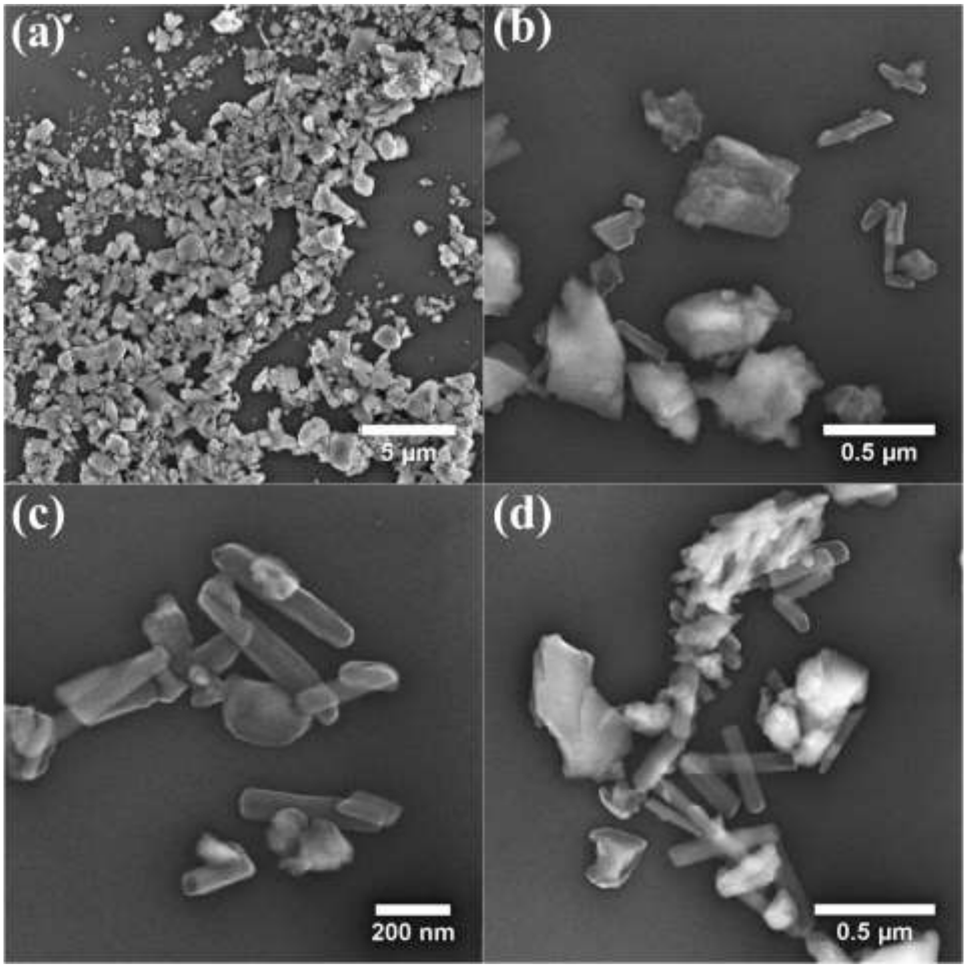

Fig. S2. (a)–(d) SEM images of some particles of the $(Nd_xY_{1-x}Al_3(BO_3)_4$ were x = 0.2, 0.6, 0.8 and 1.00 respectively.

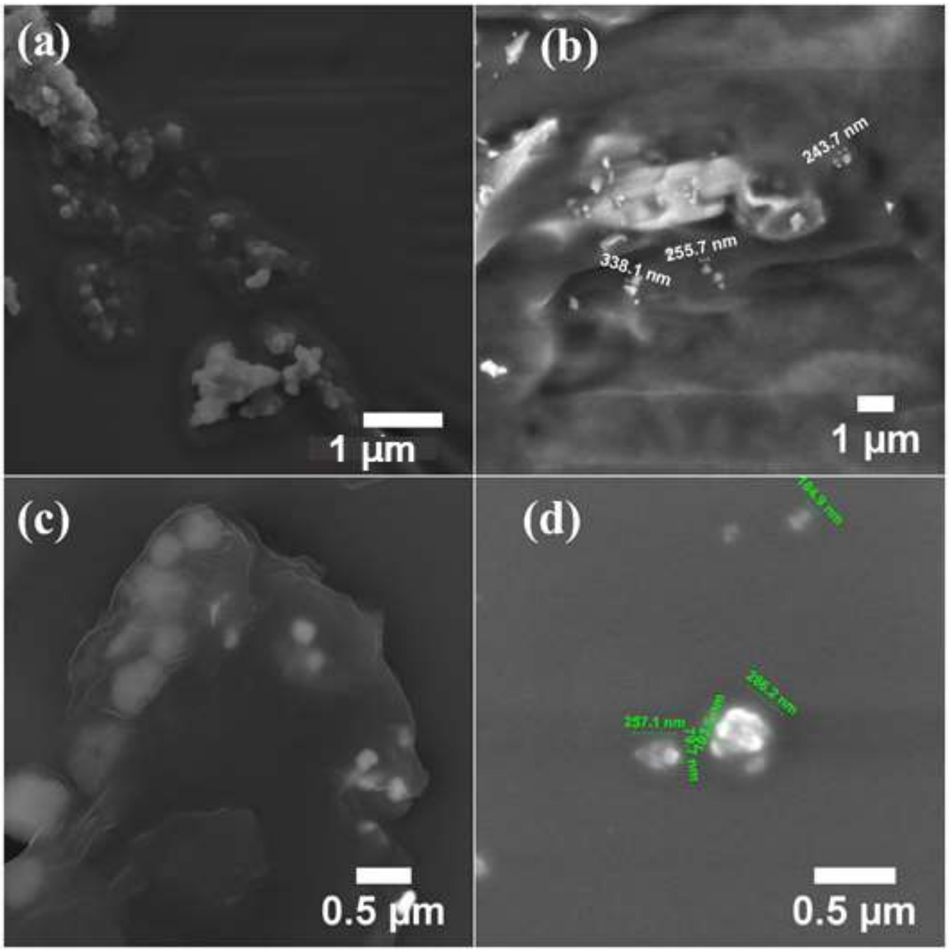

Fig. S3. SEM Images showing the morphology of filled (PC) PDMS/($Nd_xY_{1-x}Al_3(BO_3)_4$ were x = 0.2, 0.6, 0.8 and 1.0 respectively.

SI2. Thermal Studies DSC and TGA

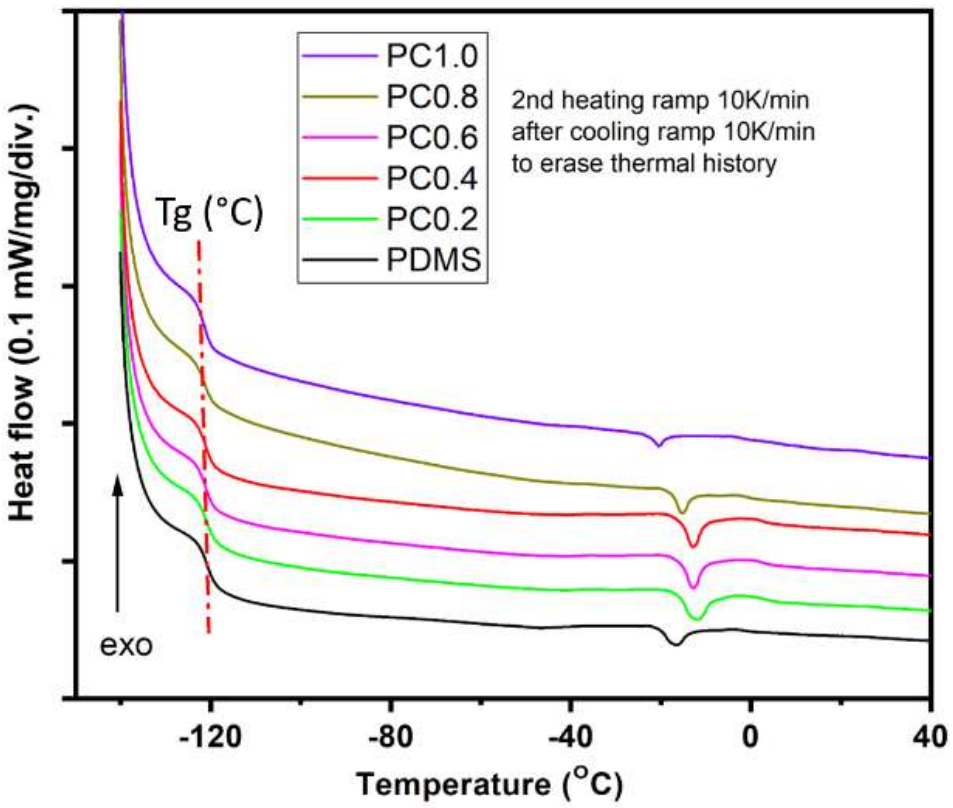

Fig. S4. DSC traces were recorded at a heating rate of 10 K/min under helium atmosphere. Offset DSC traces of the second cooling ramp from 40°C to -150°C.

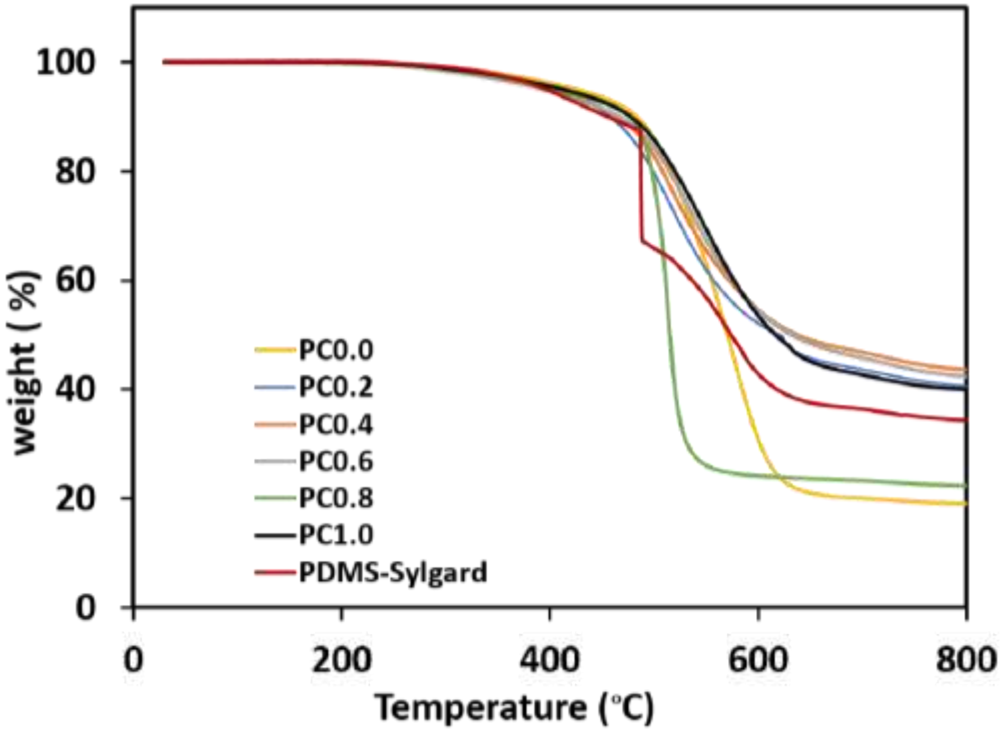

Fig. S5. Thermogravimetric analysis TGA curves of PDMS-Sylgard polymer and composites with Nd:YAB nanocomposites.

All the samples were heated up to 800°C with a constant rate of 10°C min^{-1} under 25 $mL.min^{-1}$ nitrogen streams. PDMS shows weight-loss stages which located in 220°C-450°C and 450°C-580°C which results from the PDMS degradation and hydroxyl breakage (dehydration, Evaporation of LMWS) Where all the composites give one weight losses.

S13- Random lasing emission

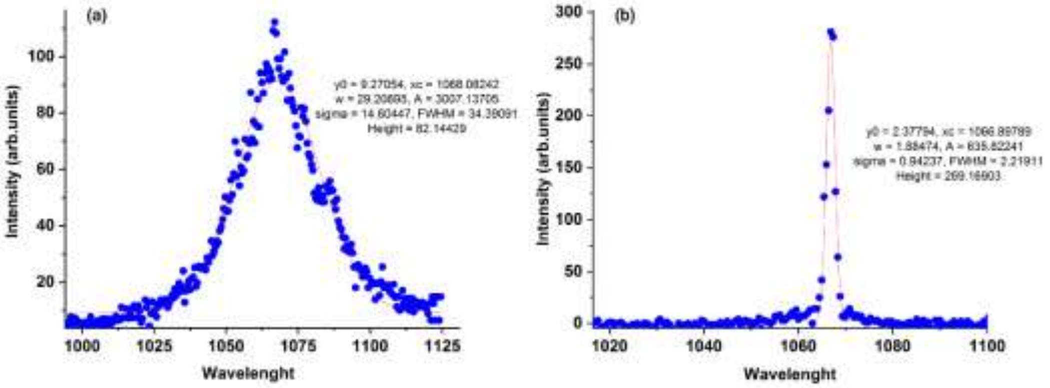

Fig. S6. FWHM obtained through Gaussian fit for ($Nd_{1.0}Al_3(BO_3)_4$) polymer composite at (a) 0.133 mJ of power and (b) 0.4 mJ of power.

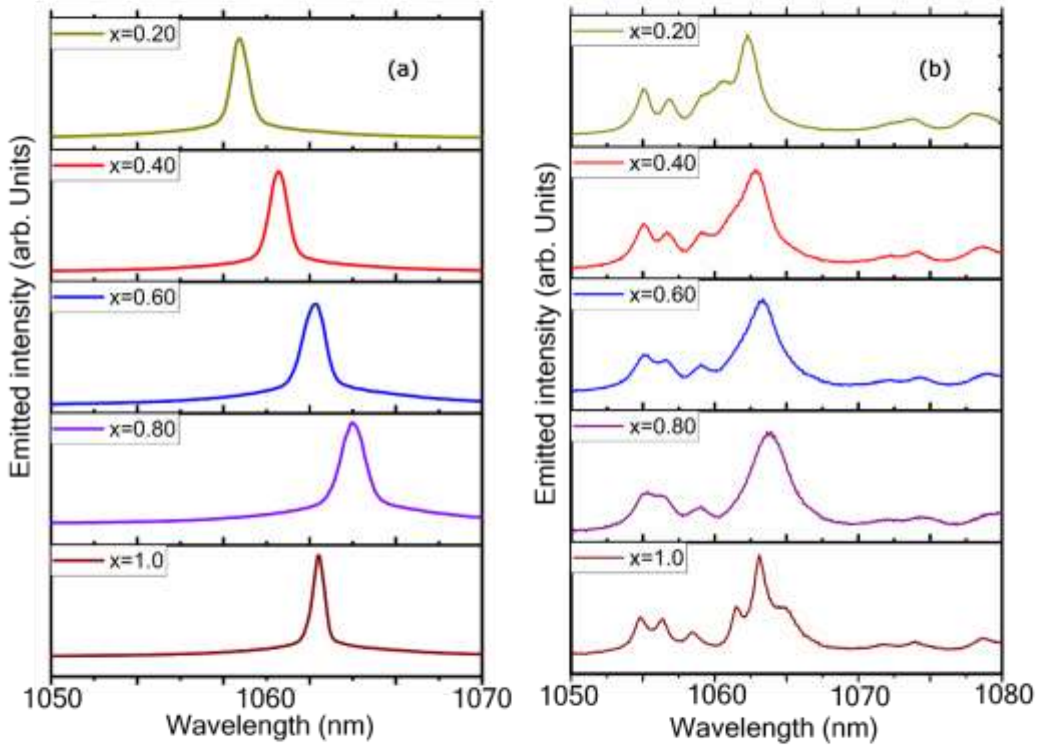

Fig. S7. Photoluminescence spectra due to the transition $^4F3/2$---$^4I11/2$ for different Nd3+ concentrations. (a) Excitation pulse energy (EPE) = 0.20 mJ (spontaneous emission) and (b) EPE = 3.0 mJ (random laser regime).

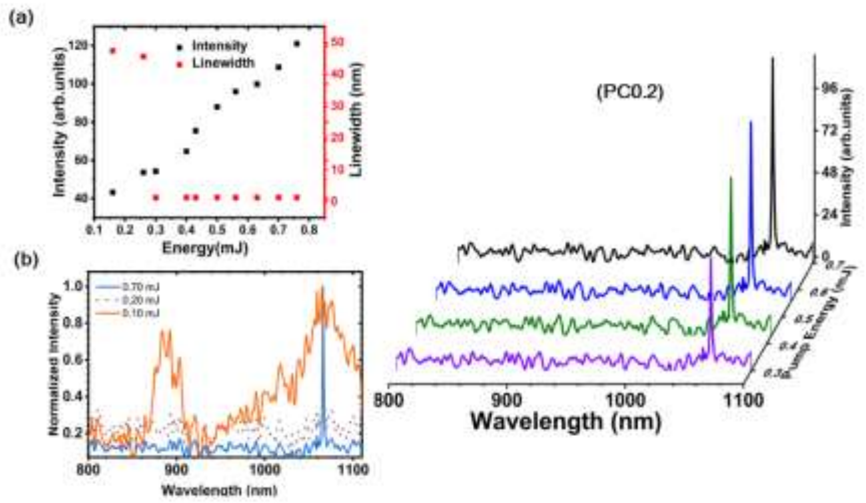

Fig. S8. The emission spectra obtained from ($Nd_{0.2}Y_{0.8}Al_3(BO_3)_4$) polymer composite. a) shows the FWHM (red circles) and maximum emission integral intensity (black circles) vs. pump energy and b) Normalized average emitted spectrum showing the linewidth reduction from below (0.2 mJ), near (1.0 mJ) and above (0.7 mJ).

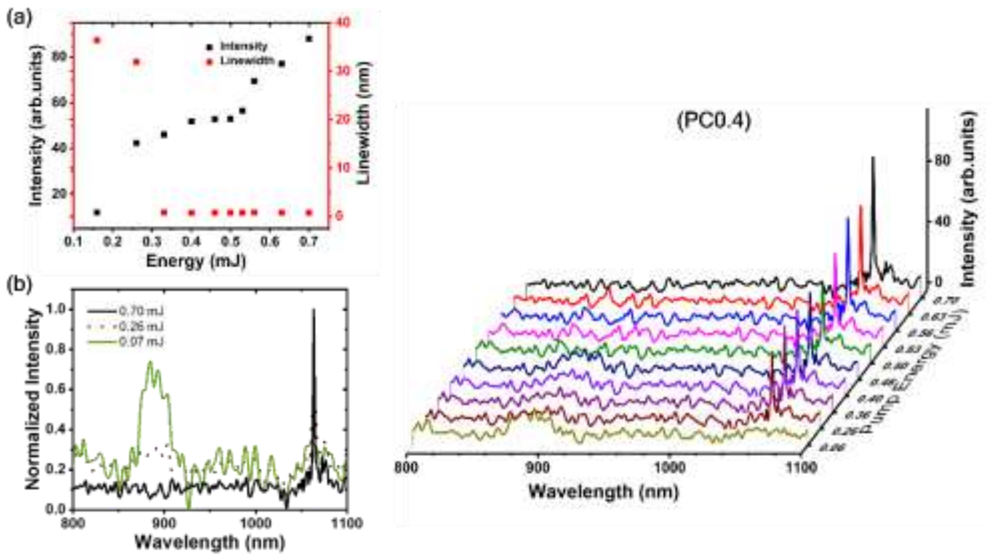

Fig. S9. a) The emission spectra obtained from ($Nd_{0.4}Y_{0.6}Al_3(BO_3)_4$) polymer composite. The threshold is 0.26 mJ. Further increase in the power, the peaks narrows to a linewidth of 0.87 ± 0.10 nm, Inset shows the FWHM (red circles) and maximum emitted intensity (black circles) dependency on pump energy of the sample ($Nd_{0.4}Y_{0.6}Al_3(BO_3)_4$) polymer composite. C) Normalized average emitted spectrum showing the linewidth reduction from below (0.0.07mJ), near (0.26 mJ) and above (0.7 mJ).

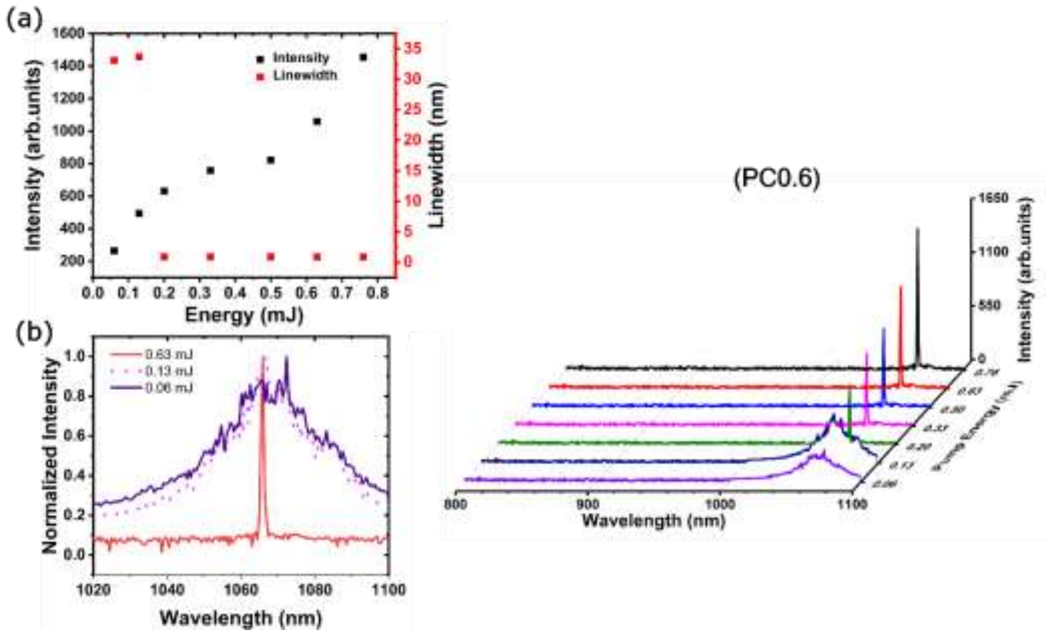

Fig. S10. a) The emission spectra obtained from ($Nd_{0.6}Y_{0.4}Al_3(BO_3)_4$) polymer composite. The threshold is 0.20 mJ. Further increase in the power, the peak narrows to a linewidth of 0.88 nm ± 0.10 nm. b) FWHM (red squares) and maximum emitted intensity (black circles) dependency vs. pump energy. c) Normalized average emitted spectrum showing the linewidth reduction from below (0.0.06 mJ), near (0.130 mJ) and above (0.63 mJ).

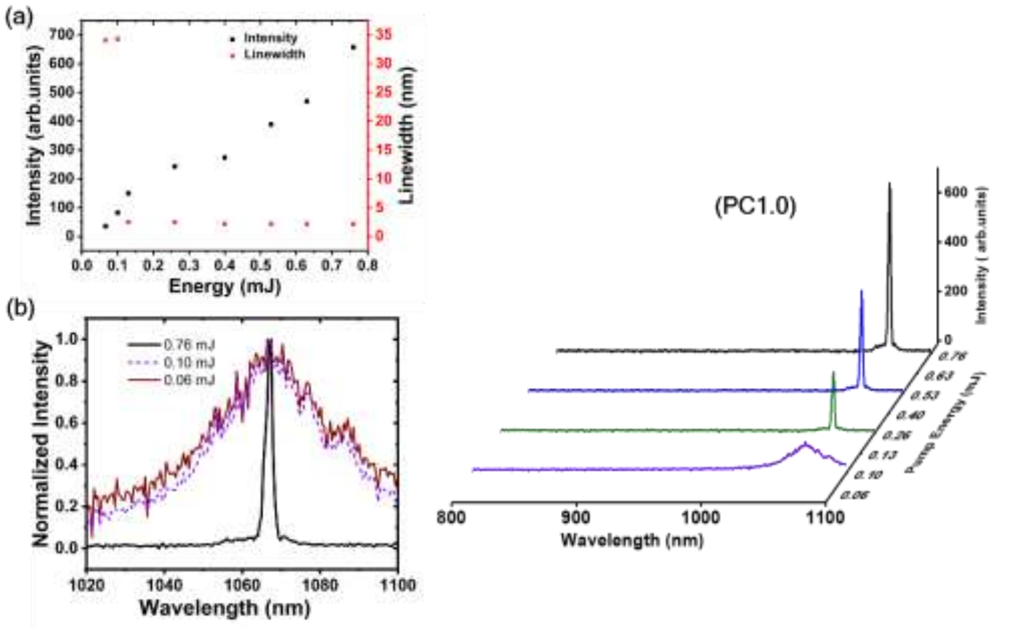

Fig. S11. a) The emission spectra obtained from ($Nd_{1.0}Al_3(BO_3)_4$) polymer composite. b) FWHM (red squares) and maximum emitted intensity (black circles) dependency vs. pump energy. c) Normalized average emitted spectrum showing the linewidth reduction from below (0.0.06 mJ), near (0.10 mJ) and above (0.76 mJ) the threshold.

SI4 Emission Lifetime

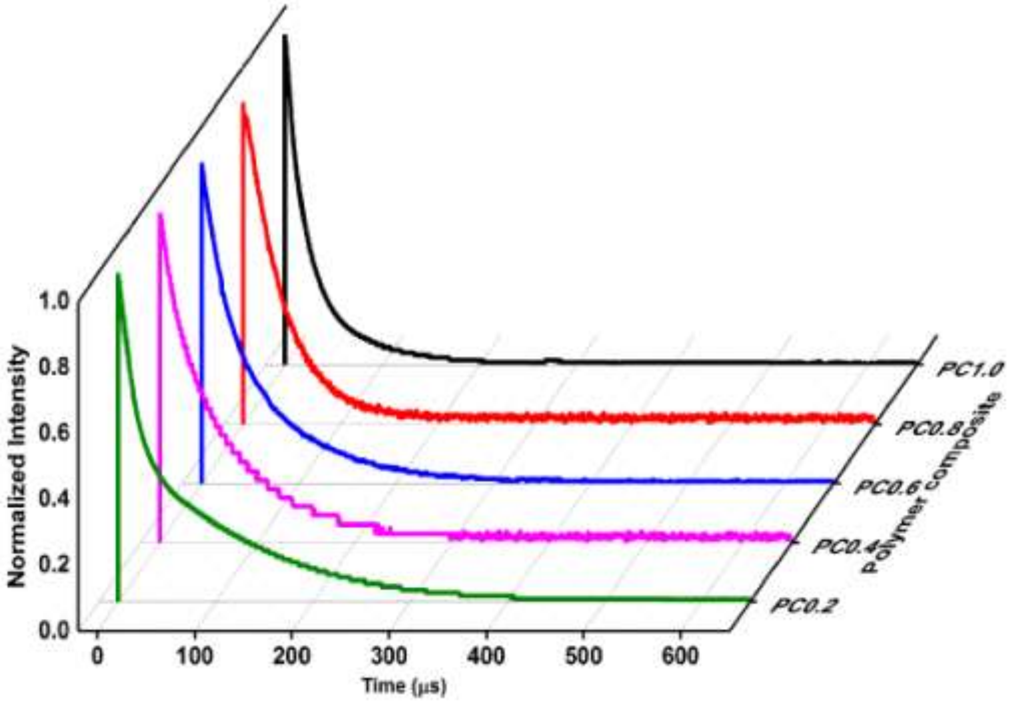

Fig. S12. The emission lifetime decay curves for all the polymer composites at an excitation wavelength of 532 nm.

SI5- Stretching studies on polymer composites.

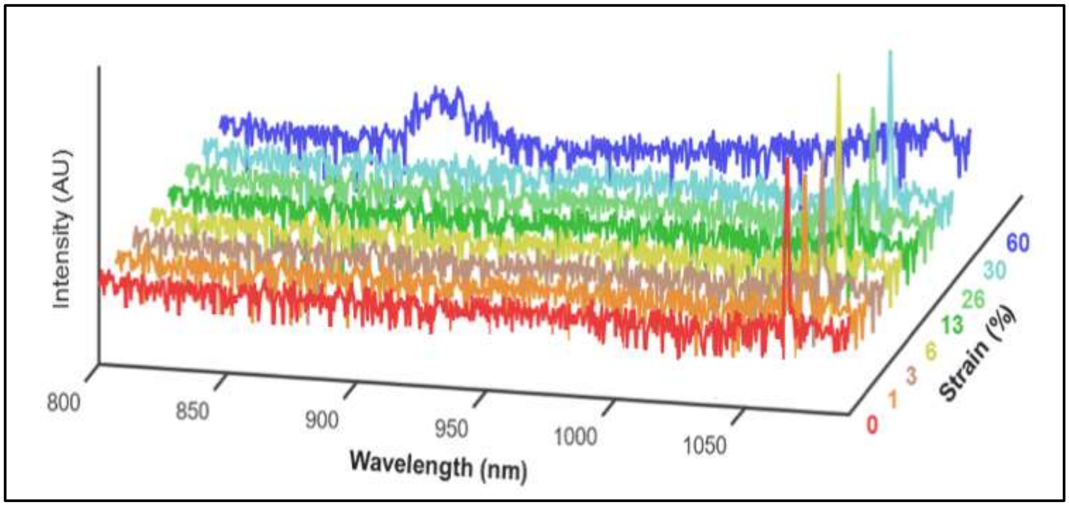

Fig. S13. The RL emission intensity as a function of strain $\Delta L/L$ (strain) applied to the PC0.2 elastomer ($Nd_{0.2}Y_{0.8}Al_3(BO_3)_4$) from 0 to 60%.

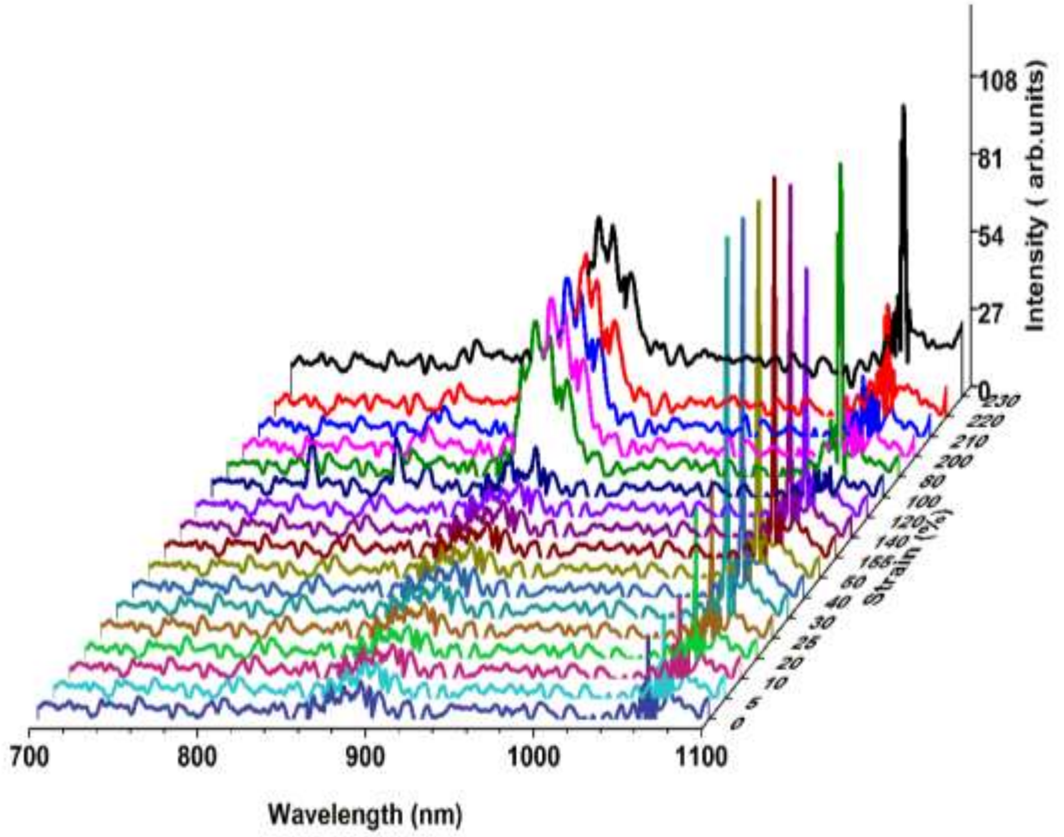

Fig. S14. The emission intensity of the PC0.4 elastomer Nd: YAB ($Nd_{0.4}Y_{0.6}Al_3(BO_3)_4$), as a function of strain ($\Delta L/L$) from a) 0 to 50 %, b) 80 to 155 % and c) 200 to 230 %.

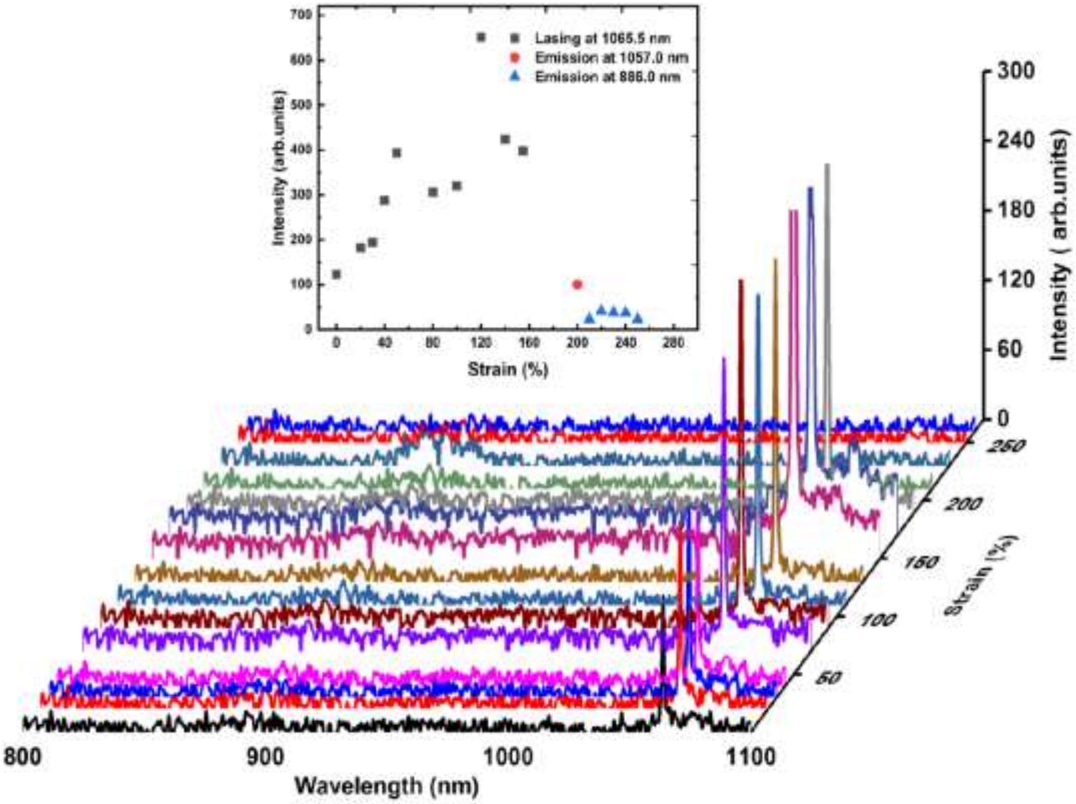

Fig. S15. The RL emission intensity as a function of ΔL/L (strain) applied to PC0.6 elastomer Nd:YAB ($Nd_{0.6}Y_{0.4}Al_3(BO_3)_4$). The lasing at 1065.95± 0.5 nm was sustained up to 270 % of strain.

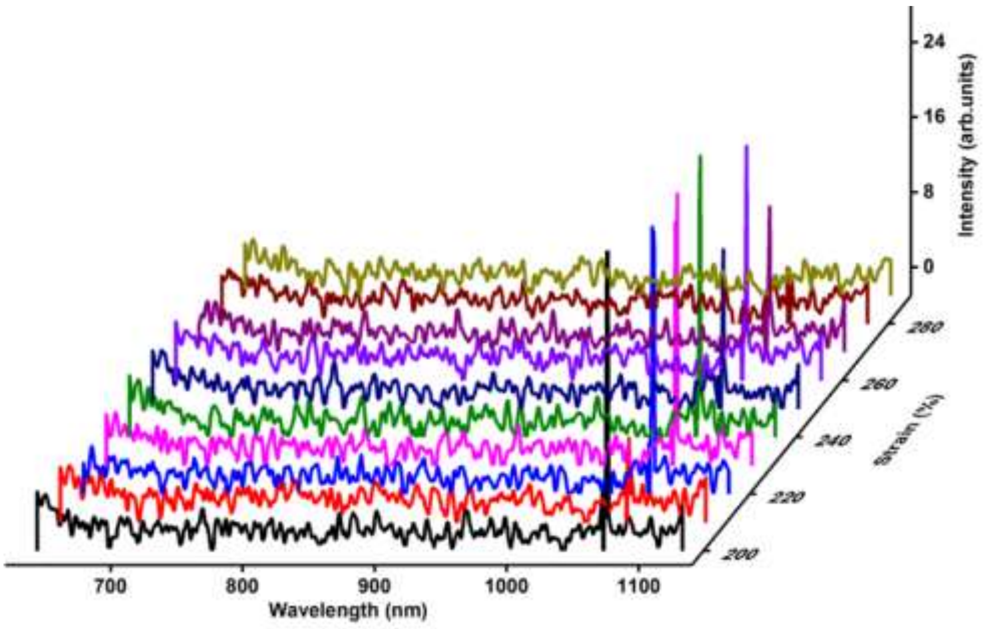

Fig. S16. The RL emission intensity as a function of ΔL/L (strain) applied to PC1.0 elastomer Nd:YAB ($Nd_{1.0}Al_3(BO_3)_4$). The lasing emission at 1066.9 ± 0.5 nm was maintained up to 300 % of stretching.

Fig. S17 shows 1,000 spectra for the PC0.8 polymer composite at each pump energy, from which the intensity fluctuations can be appreciated in the regimes below (a), around (b), just above (c), and far above (d) the RL threshold. The strong intensity fluctuations observed around and just above the threshold (Fig. 5(b) and 5(c)) suggest that the PDF of the output intensities can be described by the family of Lévy α-stable distributions, including a Lévy-like statistical regime if $0 < \alpha < 2$ and the and the Gaussian distribution if $\alpha = 2$[25-33].

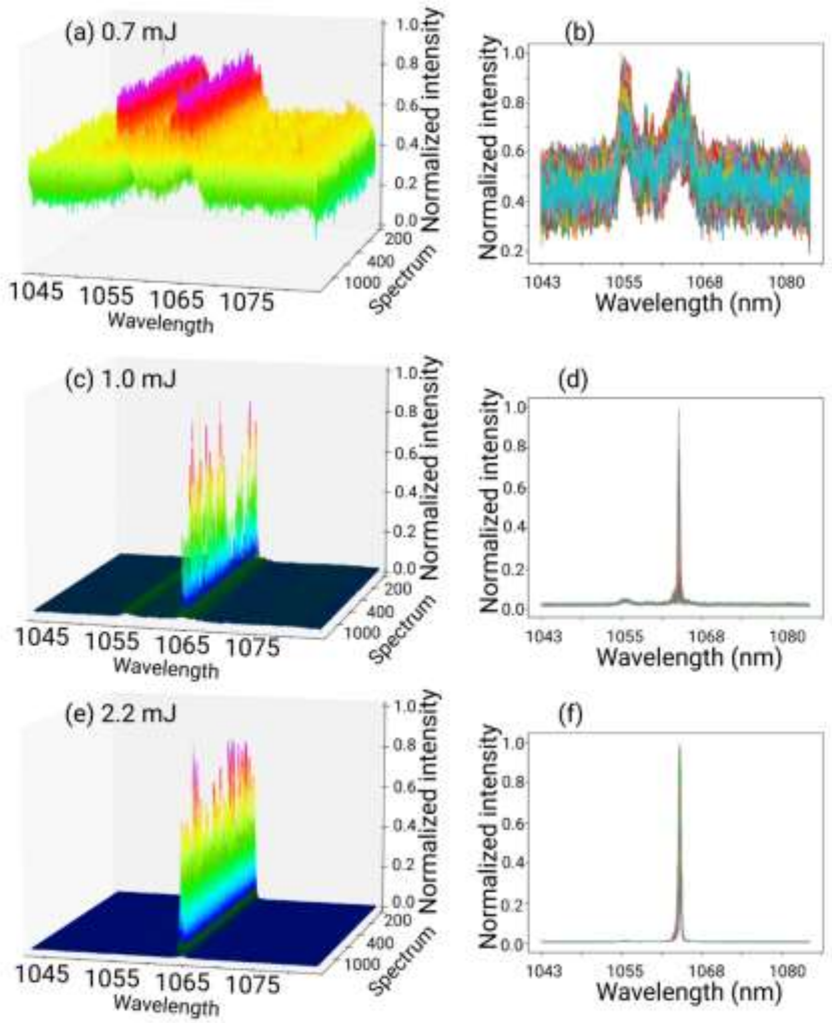

Fig. S17. 1,000 spectra for each pump energy considering the regimes below (a, b), around (c, d), and far above (e, f) the RL threshold for the polymer composite PC0.8.
